# Vitamin D Levels in Patients with Colorectal Cancer and Matched Household Members

**Published:** 2019-07-12

**Authors:** Marissa B. Savoie, Alan Paciorek, Li Zhang, Nilli Sommovilla, Alan P. Venook, Erin L. Van Blarigan, Katherine Van Loon

**Affiliations:** 1 Helen Diller Family Comprehensive Cancer Center, University of California San Francisco, USA; 2 Department of Epidemiology and Biostatistics, University of California, San Francisco, USA; 3 Division of Hematology/Oncology, Department of Medicine, University of California, San Francisco, USA; 4 Department of Medicine, Georgetown University, Washington, DC, USA

**Keywords:** Colorectal cancer, Vitamin D, Household members

## Abstract

**Background:**

Vitamin D levels, as measured by 25-hydroxyvitamin-D [25(OH) D], are inversely related to the risk of developing colorectal cancer (CRC). Given shared demographic and lifestyle factors among members of the same household, we sought to examine vitamin D levels and associated lifestyle factors in household members of CRC patients.

**Methods:**

Thirty patients with pathologically confirmed CRC were enrolled prior to oncologic therapy along with unrelated household members who were matched for age (+/− 5 years) and race. In addition to serum blood draws for 25(OH)D levels at baseline and six-month follow-up, questionnaires collected gender, vitamin use, body mass index, family history of CRC, race, dietary vitamin D, UV exposure, and exercise.

**Results:**

Median serum 25(OH) D levels were 26.8 ng/mL for CRC patients versus 27.3 for household members (P=0.89). Vitamin-D associated factors such as dietary vitamin D intake, UV exposure, gender, multivitamin use, vitamin D supplement use, and family history of CRC were not significantly different between CRC patients and paired household members (P>0.05). Household members were more likely than CRC patients to be overweight and to exercise more.

**Conclusions:**

Vitamin D levels and many associated lifestyle factors were not significantly different between CRC patients and unrelated paired household members. Given comparable vitamin D levels, further investigation into whether age-matched household members of CRC patients may be at increased risk for CRC is warranted.

## Background

Epidemiological data have substantiated that 25-hydroxyvitamin-D [25(OH)D], the physiologically active metabolite of vitamin D, is inversely related to the risk of developing colorectal cancer (CRC) [1]. Age, race, ultraviolet (UV) light exposure, dietary vitamin D consumption, vitamin D supplement use, body mass index (BMI), and blood draw season are associated with 25(OH)D levels [2]. A growing body of epidemiologic data suggests that lifestyle factors such as diet and exercise also play a role in the development of CRC [3].

In this study, we aimed to evaluate risk for vitamin D deficiency in patients with a new diagnosis of CRC and in matched household members. Our hypothesis was that patients and their household members would share common lifestyle factors associated with vitamin D status such as BMI, UV exposure, and physical activity.

## Methods

We recruited patients who were evaluated at our institution for a new diagnosis of pathologically confirmed CRC of any stage and prior to initiation of any oncologic therapy. For patients with early stage disease who underwent surgical resection of the primary tumor, patients were enrolled within eight weeks following surgical resection.

This study was approved by the UCSF Committee for Human Research (IRB Number 10–03402), and all participants provided informed consent. Measurement of 25(OH)D by clinical providers and vitamin D repletion and/or supplementation, according to individual care practices, were not restricted during the study period. A detailed description of subject enrollment, questionnaire development, and assay methodology was published previously [4].

The study was embedded in a larger study of vitamin D levels in CRC patients [4], and household members were recruited by convenience sampling for a subset of patients. Household members were invited to participate and were matched for age (±5 years) and race. Prior data have shown that with cancer patients, family or friend controls typically do not differ from matched neighborhood controls in age, BMI, tobacco use, and alcohol use [5].

CRC patients and their respective household member completed study visits on the same dates to control for seasonal variation of serum 25(OH)D levels. A baseline questionnaire administered to all participants collected gender, vitamin use, BMI, family history of CRC, race, dietary vitamin D, UV exposure, and exercise. Colon or rectal cancer in a parent or sibling was considered a family history of CRC. The dietary vitamin D questionnaire solicited consumption frequency of 13 common foods with high vitamin D contributions (milk, soy milk, yogurt, cheese, fortified orange juice, canned tuna fish, sardines, shellfish, dark meat fish, other fish, eggs, beef liver, and cold breakfast cereal) [6]. A physical activity score was calculated by self-reported frequency of performing nine common exercise activities, yielding units of activity-specific MET-hours per week. Phlebotomy samples for serum 25(OH)D assay evaluation were collected at the time of study registration and at six-month follow-up.

To compare questionnaire responses and serum 25(OH)D levels between CRC patients and household members, a likelihood ratio test fitted to a conditional logistic regression model was used for each variable. The analysis was done using SAS statistical software. Double-sided p-values <0.05 were considered statistically significant.

## Results

A total of 30 matched CRC patients and household members were enrolled; all household members were spouses or long-term partners of the paired CRC patients. Of these 30 pairs, 26 had 25(OH)D measurements performed both at baseline and six-month follow-up. Gender, multivitamin use, vitamin D supplement use, and family history of CRC were comparable among CRC patients and their household members (Table 1). CRC patients were more likely to be normal weight compared to match household members (P=0.045).

Median serum 25(OH)D level at baseline lab draw was 26.8 ng/mL for CRC patients versus 27.3 for household members (P=0.89, Table 2). At six-month follow-up 25(OH)D levels did not differ between CRC patients and household members (P=0.23). Across a variety of vitamin D associated lifestyle factors, CRC patients and their household members did not differ significantly, except that household members had higher exercise scores at baseline (P=0.02).

## Discussion

Serum 25(OH)D levels were comparable between CRC patients and household members at both baseline and six-month timepoints. Similarly, a number of lifestyle factors known to be associated with vitamin D status, including relative UV exposure and dietary vitamin D consumption, were similar between CRC patients and matched household members. Using the widely accepted vitamin D sufficiency threshold of serum 25(OH)D greater than 30 ng/mL [7,8], most participants in both groups were vitamin D insufficient or deficient. However, it has been proposed by the Institute of Medicine that the lower limit of sufficiency should be 20 ng/mL [9], which is below median serum 25(OH)D in both CRC patients and household pairs.

We did observe differences in BMI and exercise between CRC patients and unrelated household members. However, it is possible that the variables which differed, BMI and exercise, were biased by the clinical scenario following a CRC diagnosis. In consideration of the fact that 60% of CRC patients underwent tumor resection within eight weeks prior to administration of the baseline questionnaire, differences in exercise patterns amongst CRC patients versus household members were likely influenced by temporary exercise restrictions imposed during recovery from surgery. Lower BMI among CRC patients versus household members may reflect that weight loss is a common presenting symptom in patients with CRC [10].

We found that household members did not have significantly different serum 25(OH)D levels from CRC patients at baseline or six-months after treatment. Pairs were unrelated; therefore, these data likely reflect shared lifestyle factors rather than shared genetic contributions to vitamin D metabolism. CRC patients and partners were found to have numerous similarities in lifestyle factors that are known to be associated with increased risk for CRC. In light of these similarities in lifestyle, further investigation into whether unrelated household members may be at increased risk for CRC is warranted.

## Figures and Tables

**Table 1:** Summary of clinicodemographic factors.

	Total (N=60)	CRC patients (N=30)	Household members (N=30)	
	N (%)	N (%)	N (%)	P-value*
Gender	30 (50)	12 (40)	18 (60)	0.272
Female				
Male	30 (50)	18 (60)	12 (40)	
Multivitamin use at baseline †				0.244
No	38 (63)	21 (70)	17 (57)	
Yes	22 (37)	9 (30)	13 (43)	
Vitamin D supplement use at baseline †				0.403
No	37 (62)	20 (67)	17 (57)	
Yes	23 (38)	10 (33)	13 (43)	
Vitamin D supplement use at 6 months †				0.999
No	41 (68)	19 (63)	22 (73)	
Yes	5 (8)	4 (13)	1 (3)	
Unknown ‡	14 (23)	7 (23)	7 (23)	
Body-mass index, kg/m^2^				0.039
Normal [18.5, 25)	22 (37)	13 (43)	9 (30)	
Overweight [25,30)	16 (27)	5 (17)	11 (37)	
Obese >30	20 (33)	11 (37)	9 (30)	
Unknown ‡	2 (3)	1 (3)	1 (3)	
Family history of CRC				0.999
No	51 (85)	26 (87)	25 (83)	
Yes	6 (10)	3 (10)	3 (10)	
Unknown ‡	3 (5)	1 (3)	2 (7)	
Race				0.999**
Asian	6 (10)	3 (10)	3 (10)	
Caucasian	48 (80)	24 (80)	24 (80)	
Unknown‡	6 (10)	3 (10)	3 (10)	
Season of first blood draw				0.999**
Winter	25 (42)	12 (40)	13 (43)	
Spring	10 (17)	5 (17)	5 (17)	
Summer	10 (17)	5 (17)	5 (17)	
Autumn	15 (25)	8 (27)	7 (23)	

* P-value is based on likelihood ratio test by fitting a conditional logistic regression model.

** Required matched criterion for household CRC patients and household members.

‡ Unknown group not included in p-value calculation.

† Multivitamin and vitamin D supplement use was defined as self-reported consumption of a multivitamin or dedicated vitamin D supplement, respectively.

**Table 2:** Comparison of serum 25(OH)D levels and vitamin D associated lifestyle factors between CRC patients and household members.

	CRC patients		Household members		Odds ratio [95% CI]	P-value
	N	Median [range]	N	Median [range]		
Serum 25(OH)D at baseline (ng/mL)	30	26.85 [10.40,47.60]	30	27.30 [11.30,45.50]	1.0 [0.95, 1.1]	0.891
Serum 25(OH)D at 6 months (ng/mL)	26	29.10 [8.80,72.90]	26	28.05 [16.00,47.10]	1.0 [0.98, 1.1]	0.232
Serum 25(OH)D change: 6 months -baseline (ng/mL)	26	−0.60 [−9.50,51.70]	26	−1.45 [−10.40,7.00]	1.1 [.99,1.2]	0.097
Dietary vitamin D at baseline (IU/day)	30	560 [133,1981]	30	458 [167,1012]	1.0 [0.99, 1.0]	0.065
Select exercises at baseline (MET-hours/week)	30	12 [0,36]	30	19 [0,61]	0.95 [0.90, 0.99]	0.023
Relative UV exposure at baseline (Watts*hour)/meter^2^	28	5774 [159,10493]	28	4581 [1618,13252]	1.0 [0.99, 1.0]	0.935
Body-mass index, kg/m^2^	29	27.5 [19.1, 37.8]	29	27.1 [19.4, 52.3]	0.99 [0.89, 1.1]	0.938

* P-value is based on likelihood ratio test by fitting a conditional logistic regression model.
